# Zilian Ointment Promotes Diabetic Wound Healing via Akt/HIF‐1*α*–Mediated Angiogenesis

**DOI:** 10.1155/jdr/9928022

**Published:** 2026-09-24

**Authors:** Cheng Qin, Yiwen Zhang, Duhua Chen, Jin Xu, Jing Yang, Fei Liu, Qin Li, Xiang Li, Jianzhou Ye, Xuesong Yang

**Affiliations:** ^1^ Department of Dermatology, The First Affiliated Hospital of Yunnan University of Chinese Medicine, Kunming, China; ^2^ Department of Dermatology, The First Clinical Medical College, Yunnan University of Chinese Medicine, Kunming, China, ynutcm.edu.cn; ^3^ Department of Dermatology, Yunnan Provincial Hospital of Traditional Chinese Medicine, Kunming, China, yn-tcm-hospital.com; ^4^ Department of Plastic & Reconstructive Surgery, Shanghai Ninth People′s Hospital Affiliated to Shanghai Jiaotong University School of Medicine, Shanghai, China; ^5^ Department of Dermatology and Venereology, Linquan County People′s Hospital, Fuyang, China

**Keywords:** AKT/HIF-1*α* signaling, angiogenesis, diabetic wound healing, glycolysis, Zilian ointment

## Abstract

**Purpose:**

Persistent inflammation, defective angiogenesis, and metabolic dysfunction impede diabetic wound repair. Although Zilian ointment (ZLO) is clinically applied to wounds, its activity in diabetic wounds and the associated molecular basis remain incompletely understood. We therefore assessed the reparative effects of ZLO and examined the signaling events potentially involved.

**Methods:**

UHPLC‐MS was used to characterize the chemical profile of ZLO, and network pharmacology was integrated to identify potential molecular targets and pathways. A diabetic wound model was applied to assess the reparative effects of ZLO on wound closure and tissue regeneration. Histological and immunohistochemical approaches were used to evaluate angiogenesis, macrophage polarization, and the expression of hypoxia/glycolysis‐associated proteins, including HIF‐1*α*, GLUT1, LDHA, PKM2, and VEGFA. AKT/HIF‐1*α* pathway activation was further examined by western blotting.

**Results:**

ZLO‐treated db/db mice displayed a smaller residual wound area and more complete histological repair, including greater epithelial coverage, granulation tissue formation, and collagen accumulation. Treatment was accompanied by stronger CD31 and VEGFA signals, together with a macrophage profile characterized by more F4/80^+^CD206^+^ cells and fewer F4/80^+^iNOS^+^ cells. KEGG enrichment and PPI network analyses highlighted HIF‐1 signaling and AKT1, respectively. In wound sections, ZLO increased the expression of HIF‐1*α*, GLUT1, PKM2, and VEGFA. Immunoblotting further revealed an elevated p‐AKT/AKT ratio and recovery of HIF‐1*α* and VEGFA protein levels.

**Conclusion:**

ZLO facilitates diabetic wound repair, with its therapeutic effects potentially associated with AKT/HIF‐1*α*–linked metabolic regulation and enhanced angiogenic responses.

## 1. Introduction

Chronic wounds are commonly defined as wounds that fail to progress through the normal stages of healing and do not restore anatomical and functional integrity within the expected timeframe, typically persisting for more than 3 months [1]. They impose a substantial clinical and socioeconomic burden, affecting approximately 1%–2% of the population in developed countries, and are frequently associated with underlying conditions such as diabetes, vascular insufficiency, and aging [1, 2]. Among these conditions, diabetes mellitus is a major contributor to impaired wound healing [3]. Diabetes‐related wounds are heterogeneous and may arise from neuropathy, vascular insufficiency, trauma, venous disease, or skin and soft‐tissue infection [4]. Despite their differing etiologies, they commonly exhibit delayed wound closure, persistent inflammation, and increased susceptibility to secondary infection [5–7]. Although glycemic management has improved, only approximately 60% of patients attain the recommended HbA1c target of < 7.0% [8]. Therefore, therapeutic strategies that address the persistent inflammatory microenvironment of diabetic wounds, in addition to glycemic control, are needed.

Current local treatments for diabetic wounds include advanced dressings, bioactive scaffolds, negative pressure wound therapy, laser‐based interventions, and multifunctional biomaterials [9–12]. These approaches aim to control infection and exudation, reduce inflammation and oxidative stress, and promote re‐epithelialization, angiogenesis, and tissue repair [13, 14]. However, their clinical application may be constrained by cost, technical complexity, and accessibility, highlighting the need for safe and practical topical therapies with multitarget effects.

Given the multifactorial pathogenesis of diabetic wounds, traditional Chinese medicine (TCM)‐derived therapies, which contain diverse bioactive components and may regulate multiple biological processes, have attracted increasing attention [15]. Various TCM interventions, including oral formulations, topical preparations, acupuncture, and combination therapies, have demonstrated beneficial effects on diabetic wound healing [16, 17]. Among these approaches, topical formulations may provide a practical means of modulating the local wound microenvironment. Zilian ointment (ZLO), developed by Yunnan Provincial Hospital of Traditional Chinese Medicine, is a traditional topical formulation clinically utilized for various wound‐associated disorders, including burns, scalds, skin ulcers, and infections [18]. Traditionally, ZLO is considered to exert heat‐clearing, detoxifying, debridement‐promoting, and tissue‐regenerating effects. Previous studies have shown beneficial effects of ZLO in burn injury models [19, 20], patients with stage II pressure ulcers [21], and patients with diabetic foot ulcers [22, 23], suggesting its potential for the treatment of chronic wounds of diverse etiologies.

However, the active constituents and mechanisms of ZLO remain unclear. Because the final ointment is produced through a Vaseline‐based extraction process, its composition may differ from that of the original crude herbs. Therefore, systematic characterization of its chemical composition, pharmacological activity, and mechanisms of action is important for clarifying its chemical basis, improving quality control, and supporting formulation optimization.

We hypothesized that ZLO promotes diabetic wound healing by modulating the wound microenvironment and enhancing tissue regeneration. Accordingly, we characterized the chemical constituents of ZLO and evaluated its biological activities and molecular basis in a diabetic mouse model.

## 2. Methods

### 2.1. LC–MS Analysis of ZLO Components

ZLO (approval No. Dianyao Zhi Zi (Z) 20082571A) was obtained from the Yunnan Provincial Hospital of TCM. The formulation contains 12 medicinal ingredients: Arnebiae Radix, Rehmanniae Radix, Coptidis Rhizoma, Angelicae Sinensis Radix, Polygonum cuspidatum, Taraxaci Herba, Gentianae Radix, Scutellariae Radix, Phellodendri Chinensis Cortex, Violae Herba, Ampelopsis Radix, and Borneol. Chemical profiling of ZLO was conducted by Shanghai Applied Protein Technology Co. Ltd. using UHPLC–MS. An HSS T3 column (100 × 2.1 mm, 1.8  *μ*m; waters) installed on a Vanquish UHPLC platform was used for separation, with aqueous 0.1% formic acid and acetonitrile serving as the mobile‐phase solvents. The eluate was analyzed on a Q Exactive HFX mass spectrometer operated under both positive‐ and negative‐ion electrospray conditions. Compound annotation relied on exact‐mass measurements and tandem mass spectra, which were compared with an internal TCM database and the GNPS, ReSpect, and MassBank repositories.

### 2.2. Animals

Ten‐week‐old male db/db mice [B6.BKS (D)‐Lepr<sup>db</sup>/Lepr<sup>db</sup>] and age‐matched db/m mice [B6.BKS(D)‐Lepr<sup>db</sup>/+] were supplied by Cyagen (Gu′an, China; license No. SCXK (Ji) 2021‐003). The animals were maintained in a specific pathogen‐free facility at 22.0 ± 1.0°C under alternating 12‐h periods of light and darkness. Using a randomization procedure, the mice were assigned to the control (db/m), untreated diabetic model (db/db), ZLO‐treated, or Vaseline‐treated group, with eight animals in each group. Random glycemia was measured using an EZ‐7 HD glucometer (Beijing Sinomedisite Biotechnology Co. Ltd.). A glucose value exceeding 16.7 mmol/L was used as the criterion for diabetes.

### 2.3. Diabetic Wound Model and Treatment

Mice were anesthetized with isoflurane and received a subcutaneous dose of sustained‐release buprenorphine before wounding. Following depilation of the dorsum, the operative site was disinfected using 75% ethanol. A sterile biopsy punch was then used to create a 6‐mm circular excision extending through the full thickness of the dorsal skin. The cutaneous wounds in the ZLO and Vaseline groups were topically treated once daily with either ZLO ointment or Vaseline. Vaseline was used as the vehicle control because it is the ointment base of ZLO. Each 1 g of ZLO ointment corresponds to 0.3 g of crude herbal material. Based on the clinically used topical dosage of ZLO normalized according to wound surface area, ZLO was applied once daily at a dose of 100 mg/cm^2^ of wound area. For the 6‐mm‐diameter wounds used in this study, this corresponded to approximately 28.3 mg of ZLO ointment per wound per day, equivalent to approximately 8.5 mg of crude herbal material. The Vaseline‐treated wounds received the same quantity of vehicle as that used for ZLO administration, whereas no topical treatment was given to the untreated db/m control or diabetic model groups. Wound images and body‐weight data were recorded at baseline and on postinjury days 3, 7, 10, and 14. Wound areas were quantified with ImageJ, and closure was calculated as 100 × (*A*_0_ − *A*_*t*_)/*A*_0_, where *A*_0_ represents the wound area on Day 0 and *A*_*t*_ denotes the area measured at each subsequent time point.

### 2.4. Histological and Immunofluorescence Staining

On day 14 after injury, full‐thickness dorsal tissue blocks encompassing the wound bed, periwound skin, and subjacent muscle were excised and immersed in 4% paraformaldehyde. After paraffin processing, the samples were cut into 5‐*μ*m sections and mounted on glass slides. Hematoxylin and eosin staining was used to examine overall tissue architecture, whereas Masson′s trichrome staining was applied to visualize collagen accumulation.

For immunohistochemistry, paraffin sections were dewaxed and subjected to antigen unmasking. Nonspecific binding was minimized by treatment with 5% bovine serum albumin. The slides were subsequently exposed at 4°C overnight to the following primary antibodies: *α*‐SMA (1:800, GB111364), VEGFA (1:250, GB15165), CD31 (1:500, GB150217), HIF‐1*α* (1:1000, GB151339), GLUT1 (1:2000, GB153495), PKM2 (1:1000, GB15392), and LDHA (1:500, GB150342). Sections were subsequently treated with HRP‐conjugated goat antirabbit or goat antimouse IgG secondary antibodies (1:200; G1215 or G1214). Immunoreactive signals were visualized with DAB, followed by hematoxylin nuclear staining.

For immunofluorescence staining, sections were exposed to antibodies against F4/80 (1:500, GB113373), CD206 (1:500, GB113497), and iNOS (1:500, GB11119), followed by Alexa Fluor 488‐ or Cy3‐conjugated goat antirabbit IgG (1:500, GB25303 or GB21303) incubation for 1 h at room temperature in darkness. Nuclei were labeled with DAPI (G1012) for 8 min. Microscopic images were collected and quantified using ImageJ. Evaluation of staining, image analysis, and quantitative measurements were performed in a blinded manner. Neoepithelialization was calculated as the proportion of neoepithelium length relative to total wound length: (neoepithelium length/total wound length) × 100*%* [24].

### 2.5. Network Pharmacology

Since ZLO is a topical drug, active compounds were selected based on their drug‐likeness (DL) values, which are commonly used to evaluate solubility and chemical stability [25]. Compound‐related targets were collected from HERB, BATMAN‐TCM, TCMSP, and CTD databases, whereas diabetic wound‐associated genes were obtained from GeneCards and OMIM. The intersection of compound and disease targets was used for protein–protein interaction (PPI) network construction in STRING. Hub targets were identified based on network topology analysis using the igraph package in R, including degree, betweenness, and closeness centrality. The top‐ranked targets were subjected to GO and KEGG enrichment analyses using DAVID, and enriched terms were visualized with R software.

### 2.6. Western Blot (WB) Analysis

Wound tissue lysates were prepared in RIPA buffer containing protease and phosphatase inhibitors. Equal protein amounts were analyzed by western blotting using antibodies against HIF‐1*α* (1:500, GB154936), VEGFA (1:500, GB11034B), AKT (1:1000, GB15689), p‐AKT (Ser473, 1:1000, GB150002), and *β*‐actin (1:5000, GB15003). HRP‐conjugated goat antirabbit IgG (1:5000, GB23303) was applied as the secondary antibody. Protein signals were visualized by chemiluminescence and quantified using ImageJ. Relative protein levels were normalized to *β*‐actin, whereas p‐AKT was normalized to total AKT. All antibodies used in this study were purchased from Wuhan Servicebio Technology Co. Ltd. (Wuhan, Hubei, China).

### 2.7. ELISA

Wound tissue levels of TNF‐*α* and IL‐1*β* were determined by ELISA using commercially available mouse cytokine kits (JYM0218Mo and JYM0531Mo‐T, respectively) from Wuhan Colorful Gene Biological Technology Co. Ltd. (Wuhan, Hubei, China).

### 2.8. Statistics

Data are shown as mean ± SEM. Statistical calculations were performed in GraphPad Prism (Version 9.5.0). Longitudinal measurements were analyzed by two‐way ANOVA combined with Sidak′s correction. Differences among multiple groups at single time points were assessed using one‐way ANOVA with Tukey′s multiple comparisons test. A value of *p* < 0.05 was considered statistically significant.

## 3. Result

### 3.1. Chemical Composition Profiling of ZLO

**Figure 1:**
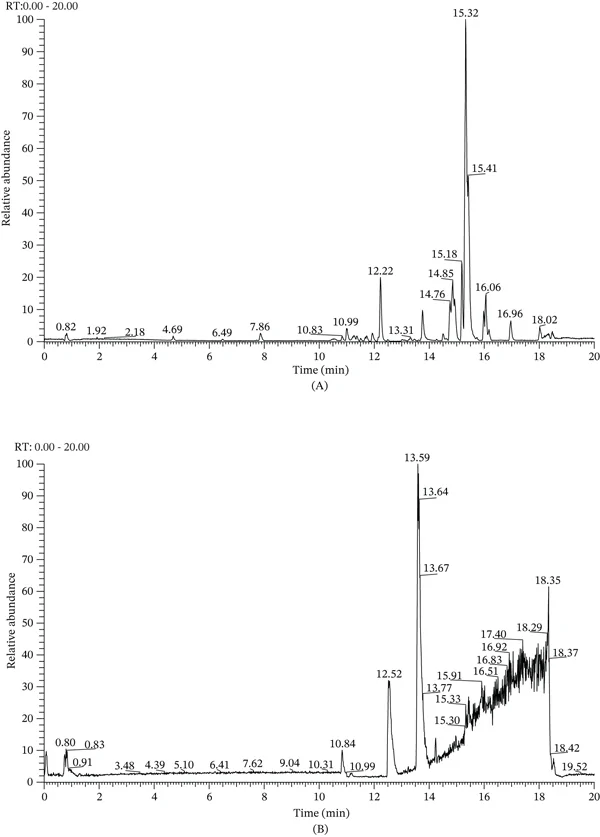
Chemical composition profiling of ZLO by UPLC‐HRMS. (A) Positive ion mode. (B) Negative ion mode.

The total ion chromatograms of ZLO obtained in the positive (ESI+) and negative (ESI−) ion modes are shown in (Figure 1A,B) and 34 major compounds identified across both ion modes are summarized in Table 1. The major compound classes identified were shikimates and phenylpropanoids (31%), flavonoids (11%), polyketides (11%), saccharides (11%), fatty acids (8%), fatty acids and conjugates (8%), and others (20%).

**Table 1:** LC‐MS/MS characterization of representative herbal‐derived compounds in ZLO.

No.	Fragment ion (m/z)	RT min	Formular	Adduct	Identification
1	193.1225	11.37	C12H16O2	[M + H]+	Senkyunolide
2	191.1067	12.23	C12H14O2	[M + H]+	Ligustilide A
3	104.1074	0.81	[C5H14NO]+	[M]+	Choline cation
4	147.0441	5.98	C16H14O4	[M + H‐C7H8O2]+	Isoimperatorin
5	153.0541	4.69	C8H8O3	[M + H]+	Vanillin
6	167.0339	13.76	C8H4O3	[M + H + H2O]+	Phthalic anhydride
7	170.0964	17.96	C12H11N	[M + H]+	Diphenylamine
8	185.1148	5.74	C10H18O4	[M + H‐H2O]+	Sebacic acid
9	191.1067	11.51	C12H14O2	[M + H]+	Butylphthalide
10	195.0653	7.88	C10H10O4	[M + H]+	Caffeic acid methylester
11	245.1173	12.32	C15H16O3	[M + H]+	Osthole
12	247.0941	6.51	C14H14O4	[M + H]+	Marmesin
13	247.0966	12.11	C20H24O9	[M + H‐C6H10O5]+	Torachrysone 8‐glucoside
14	267.1719	13.35	C12H27O4P	[M + H]+	Tributyl phosphate
15	277.086	13.23	C18H12O3	[M + H]+	Tanshinone A
16	285.0757	11.14	C22H20O11	[M + H‐C6H8O6]+	Oroxyloside
17	356.1833	12.5	C21H25NO4	[M + H]+	Tetrahydropalmatin
18	365.1053	0.87	C12H22O11	[M + Na]+	Sucrose
19	375.1073	10.83	C19H18O8	[M + H]+	Neobaicalein
20	381.206	13.76	C24H28O4	[M + H]+	Levistilide A
21	397.0894	10.83	C19H18O8	[M + Na]+	Casticin
22	413.2665	16.33	C24H38O4	[M + Na]+	Diisooctyl phthalate
23	439.357	15.44	C30H48O3	[M + H‐H2O]+	Ursolic acid
24	269.0459	12.56	C15H10O5	[M‐H]‐	Emodol
25	133.0136	0.95	C4H6O5	[M‐H]‐	(S)‐Malate
26	169.0139	1.96	C22H18O11	[M‐H‐C15H12O6]‐	(‐)‐Gallocatechin 3‐gallate
27	181.0714	0.83	C6H14O6	[M‐H]‐	Dulcitol
28	187.0971	6.29	C9H16O4	[M‐H]‐	Azelaic acid
29	191.0205	1.19	C6H8O7	[M‐H]‐	Citric acid
30	283.0616	11.17	C16H12O5	[M‐H]‐	Oroxylin
31	283.0616	10.86	C16H12O5	[M‐H]‐	Wogonin
32	315.2545	13.24	C17H34O2	[M + HCO2]‐	Methyl palmitate
33	503.1628	0.87	C18H32O16	[M‐H]‐	Gentianose
34	665.2165	0.83	C24H42O21	[M‐H]‐	Stachyose

Abbreviations: adduct, detected ion adduct form; No., compound number; RT (min), retention time.

### 3.2. ZLO Promotes Wound Healing in Healthy and Diabetic Mice

After chemical profiling of ZLO, its therapeutic activity was investigated in a db/db mouse model of diabetic wound repair (Figure 2A). Relative to db/m mice, db/db mice displayed compromised healing capacity, with a marked delay in wound closure. In particular, db/db mice exhibited delayed wound closure and sustained wound enlargement during the early stage, resulting in a prolonged healing period, consistent with previous reports (Figure 2D,E) [26]. By day 14, the control animals exhibited near‐complete wound closure, whereas substantial residual defects were still evident in the other groups. Compared with untreated db/db mice, topical administration of either Vaseline or ZLO promoted wound repair and reduced inflammatory manifestations at the wound site. Importantly, ZLO treatment resulted in a greater improvement in wound closure compared with Vaseline treatment at both Day 7 and Day 14 (*p* < 0.05). Moreover, wounds receiving ZLO exhibited enhanced tissue filling within the wound bed.

Figure 2ZLO promotes wound healing in diabetic mice. (A) Schematic diagram of the wound model and ZLO treatment. (B) Body weight at each treatment time point, (C) random blood glucose levels before treatment. (D) Wound closure rates at the indicated time points, (E) representative images and heatmaps. (F) Neoepithelium length rate, (G) collagen volume fraction, (H) H&E and MT staining images of the wounded skin sections on Day 14. (B–D, *n* = 8; F–G, *n* = 4,  ^∗^ *p* < 0.05,  ^∗∗^ *p* < 0.01,  ^∗∗∗^ *p* < 0.001).

**Figure:**
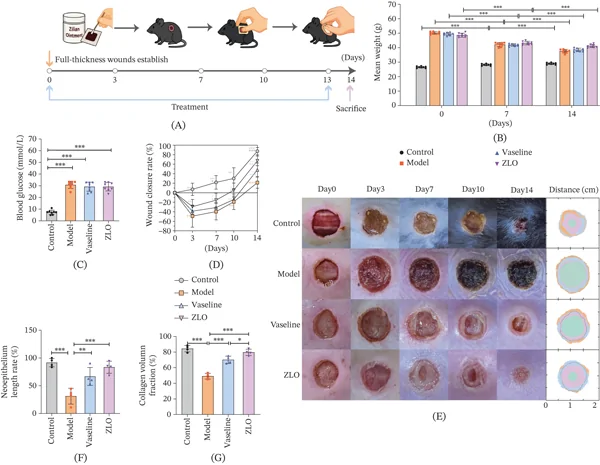

**Figure:**
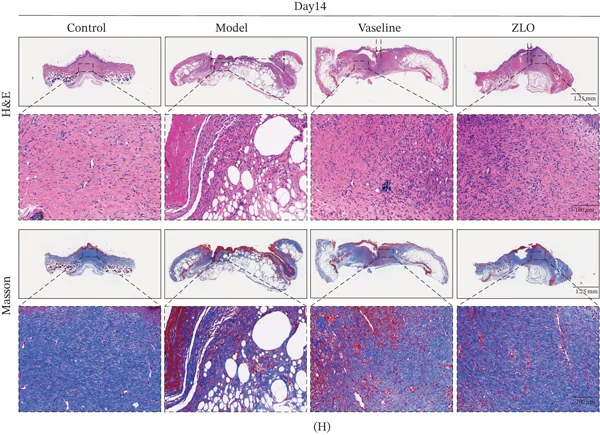

To characterize the tissue‐level response to ZLO, wound sections were subjected to H&E and Masson′s trichrome staining for evaluation of overall morphology and collagen deposition (Figure 2F–H). The control group showed better healing, with the epithelialization rate approaching complete closure by day 14. Compared with the model and Vaseline groups, ZLO‐treated wounds exhibited markedly less inflammatory cell infiltration and edema. Both the ZLO and Vaseline groups exhibited increased re‐epithelialization, enhanced granulation tissue formation, greater collagen deposition, and more advanced tissue remodeling. Conversely, the model group exhibited persistent inflammatory cell infiltration, a wider residual wound gap, and immature collagen deposition. Quantitative analysis revealed marked improvements in epithelial coverage and collagen accumulation following ZLO treatment. The epithelialization rate reached 83.28 ± 10.90% in the ZLO group, which was substantially greater than that observed in the model group (31.20 ± 14.37%, *p* < 0.001). Similarly, collagen volume fraction increased to 79.58 ± 4.08% after ZLO administration, exceeding the values measured in both the model group (49.04 ± 3.66%, *p* < 0.001) and the Vaseline group (70.10 ± 4.63%, *p* < 0.01).

### 3.3. ZLO Enhances Angiogenesis and Promotes M2‐Associated Macrophage Polarization

**Figure 3:**
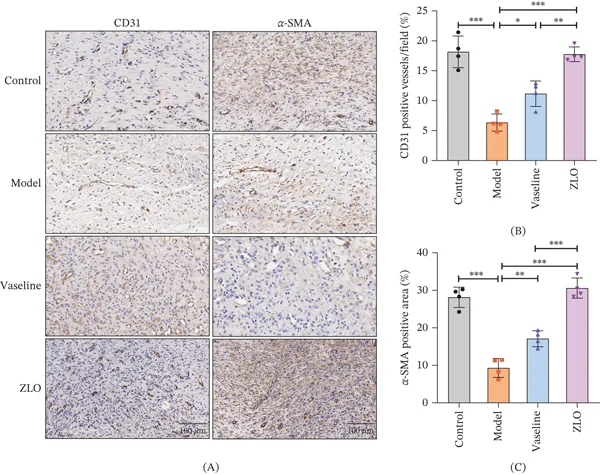
ZLO promotes vascularization and tissue remodeling during diabetic wound repair. (A) Representative immunohistochemical images showing CD31 and α‐SMA expression among the indicated groups. (B,C) Semiquantitative analysis of (B) the CD31‐positive area and (C) α‐SMA‐positive area in the indicated groups. (*n* = 4;  ^∗^ *p* < 0.05,  ^∗∗^ *p* < 0.01,  ^∗∗∗^ *p* < 0.001).

Immunohistochemical analysis was performed to evaluate CD31 and *α*‐SMA expression following ZLO treatment. Both ZLO and Vaseline treatments increased angiogenesis (CD31) and collagen deposition (*α*‐SMA) (Figure 3A), with the ZLO group showing a more significant effect compared with the Vaseline group.

**Figure 4:**
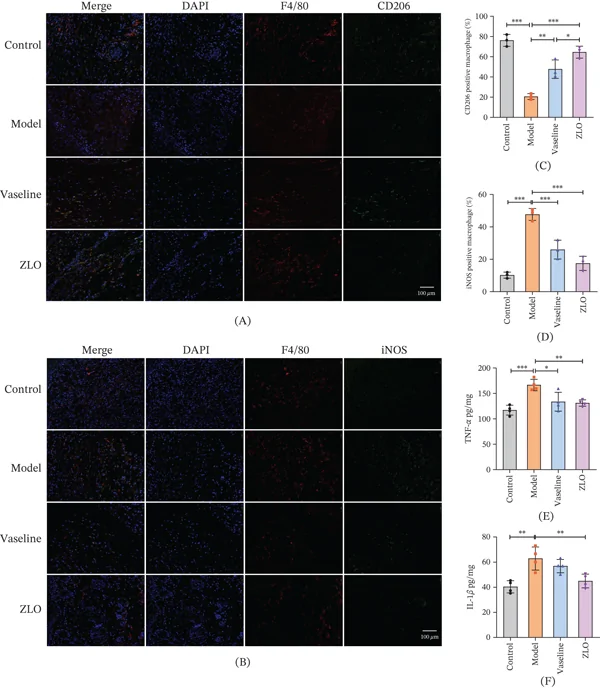
ZLO modulates macrophage polarization and inflammatory responses in diabetic wounds. (A) Representative immunofluorescence images of F4/80 (red)/CD206 (green) double staining in wound tissues. (B) Representative images of F4/80 (red)/iNOS (green) staining. (C, D) Quantification of CD206^+^ and iNOS^+^ macrophage populations based on immunofluorescence analysis. (E, F) TNF‐*α* and IL‐1*β* levels determined in wound tissue homogenates by ELISA. (A–D, *n* = 3; E–F, *n* = 4;  ^∗^ *p* < 0.05,  ^∗∗^ *p* < 0.01,  ^∗∗∗^ *p* < 0.001).

In diabetic wounds, macrophage phenotype switching is frequently disrupted, leading to prolonged inflammatory activation and an imbalance toward the M1 phenotype. On day 14, we evaluated macrophage polarization and associated cytokine levels to assess the local immune response. Immunofluorescence quantification showed that both ZLO and Vaseline treatments increased the proportion of F4/80^+^CD206^+^ M2 macrophages relative to the model group, whereas ZLO produced a greater increase than Vaseline treatment (Figure 4A,C). In contrast, both ZLO and Vaseline treatments decreased the proportion of M1 macrophages (F4/80^+^iNOS^+^) compared with the model group, accompanied by enhanced M2‐associated macrophage polarization (Figure 4B,D). Cytokine quantification revealed reduced TNF‐*α* production after ZLO or Vaseline administration, whereas the decrease in IL‐1*β* reached statistical significance only in the ZLO‐treated wounds (Figure 4E,F).

### 3.4. Network Pharmacology Analysis of ZLO

**Figure 5:**
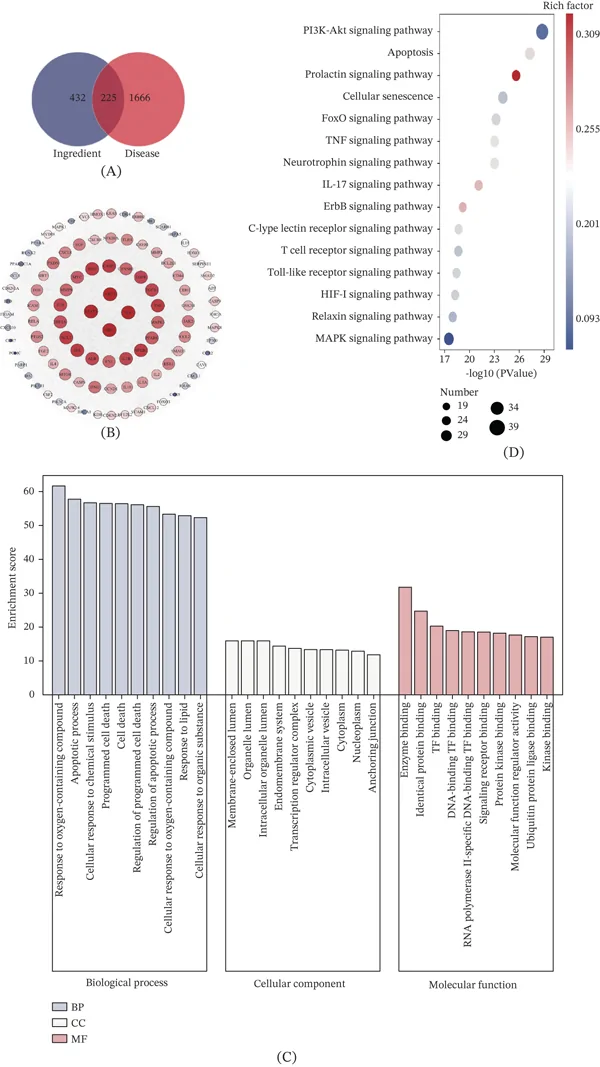
Bioinformatic exploration of potential targets and pathways associated with ZLO. (A) Overlapping targets identified between ZLO‐related compounds and diabetic wound‐associated genes. (B) Protein–protein interaction network of candidate targets. (C) GO enrichment and (D) KEGG pathway analysis of the identified targets.

To further elucidate the underlying regulatory mechanisms, DL ≥ 0.18 was applied for preliminary screening, considering that ZLO is a topical formulation. As a result, 15 active components were identified, including isoimperatorin (0.23), marmesin (0.18), torachrysone 8‐glucoside (0.53), tanshinone A (0.36), tetrahydropalmatine (0.64), sucrose (0.23), neobaicalein (0.44), levistilide A (0.82), casticin (0.44), ursolic acid (0.75), emodol (0.24), (−)‐gallocatechin 3‐gallate (0.77), oroxylin (0.23), wogonin (0.23), and stachyose (0.59), with the numbers in parentheses representing their respective DL values. A total of 657 potential targets for these active components were identified through the HERB, BATMAN‐TCM, TCMSP, and CTD databases. By intersecting the predicted targets with 1891 diabetic wound–related genes obtained from the GeneCards and OMIM databases, we identified 225 overlapping genes (Figure 5A, Table S1).

All 225 overlapping targets between the compound‐related and disease‐related datasets were uploaded to the STRING database. These proteins were used to construct the PPI network, and the top 100 targets—ranked by degree—were subsequently visualized in Cytoscape 3.9.1 (Figure 5B). The 20 genes exhibiting the highest degree values were designated as the hub targets: *TP53* (degree = 100), *AKT1* (degree = 99), *STAT3* (degree = 82), *IL6* (degree = 80), *CTNNB1* (degree = 77), *CASP3* (degree = 74), *BCL2* (degree = 73), *SRC* and *TNF* (degree = 72), *MYC* (degree = 68), *MAPK3* (degree = 66), *JUN* (degree = 65), *NFKB1* (degree = 64), *FN1* (degree = 63), *MMP9* and *ESR1* (degree = 60), *MAPK1* (degree = 59), *CCND1* (degree = 53), *INS* and *MAPK8* (degree = 49).

The GO analysis revealed that the targets were predominantly involved in biological processes such as response to oxygen‐containing compounds, apoptotic process, cellular response to chemical stimulus, and programmed cell death, indicating their roles in stress responses and cell death regulation. With respect to cellular component annotation, the predicted targets were predominantly enriched in membrane‐bounded lumens, including organelle lumens and the endomembrane system, suggesting potential involvement in intracellular compartmental processes. In addition, significant enrichment was observed in cytoplasmic vesicles and anchoring junctions, suggesting potential roles in vesicular transport and cell–cell interactions. The molecular functions of the targets included enzyme binding, transcription factor binding, and signaling receptor binding (Figure 5C). KEGG enrichment analysis was further performed after removing pathways classified as human disease‐related, and the 15 most significantly enriched pathways were selected for visualization (Figure 5D). These pathways mainly included PI3K–Akt, TNF, IL‐17, HIF‐1, and MAPK signaling pathways, as well as apoptosis‐related processes. These pathways are closely associated with inflammatory regulation, cell survival, angiogenesis, and tissue regeneration, indicating that ZLO may influence diabetic wound repair through these biological processes.

### 3.5. ZLO Activates the AKT/HIF‐1*α* Signaling Pathway to Enhance Glycolysis and Angiogenesis

To further investigate whether hypoxia‐related signaling and glycolytic pathways were involved in ZLO‐mediated diabetic wound healing, immunohistochemical staining was performed on wound tissues (Figure 6A–F). Immunohistochemical quantification revealed a marked decrease in HIF‐1*α* H‐score in the model group relative to the control group. Conversely, ZLO treatment significantly increased HIF‐1*α* expression compared with both the model and Vaseline groups (*p* < 0.05). Similarly, GLUT1 and LDHA protein levels were reduced in the model group, indicating downregulation of glycolysis‐associated molecular signatures in diabetic wounds. Following ZLO administration, GLUT1 and LDHA expression were recovered compared with the model group (*p* < 0.05), whereas Vaseline treatment did not produce a significant elevation of these markers. Furthermore, the mean H‐score of PKM2 in the ZLO group was significantly higher than that in the model group (*p* < 0.05), suggesting enhanced glycolysis‐associated activity in wound tissues. VEGFA levels were markedly elevated following ZLO treatment compared with both the model and Vaseline groups (*p* < 0.01), indicating enhanced angiogenic responses.

Figure 6Alterations of hypoxia‐ and glycolysis‐associated markers following ZLO treatment. Representative immunohistochemical images of (A) HIF‐1*α* ,(B) GLUT1, (C) LDHA, (D) PKM2, and (E) VEGFA in wound sections from different groups. (F) Quantitative evaluation of marker expression based on H‐score analysis. (*n* = 4;  ^∗^ *p* < 0.05,  ^∗∗^ *p* < 0.01,  ^∗∗∗^ *p* < 0.001).

**Figure:**
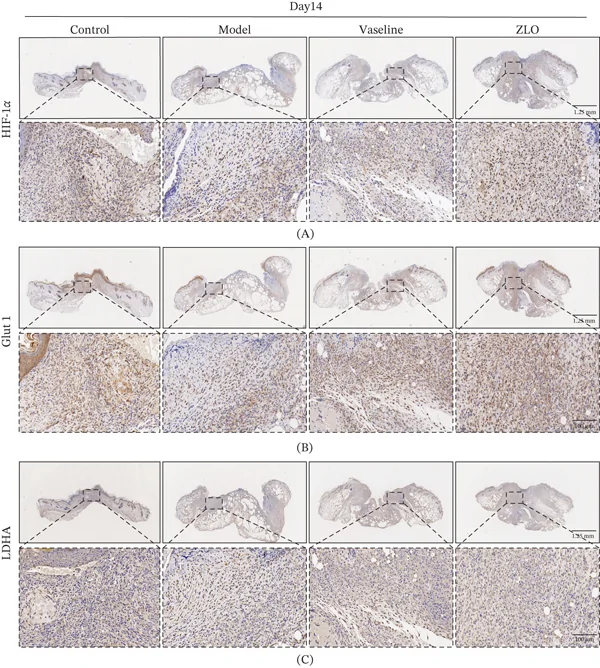

**Figure:**
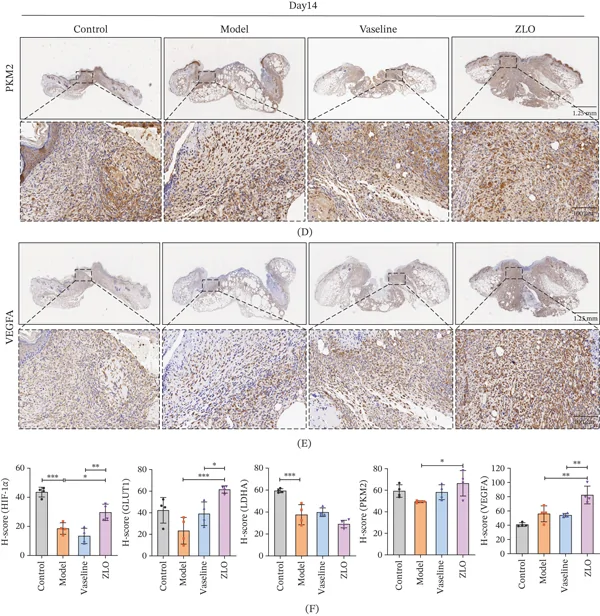

**Figure 7:**
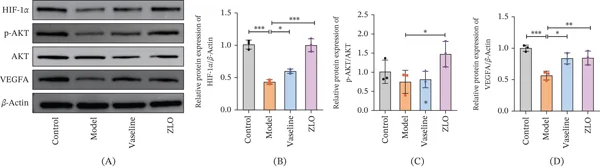
ZLO modulates AKT/HIF‐1*α*‐associated signaling proteins in diabetic wounds. (A) Immunoblot analysis of HIF‐1*α*, AKT, p‐AKT, and VEGFA expression in wound tissues. (B–D) Densitometric analysis of protein bands quantified using ImageJ software. (*n* = 3;  ^∗^ *p* < 0.05,  ^∗∗^ *p* < 0.01,  ^∗∗∗^ *p* < 0.001).

Pathway enrichment analysis identified the PI3K/AKT and HIF‐1 signaling pathways as potential signaling pathways underlying ZLO‐mediated diabetic wound healing. Among the predicted hub targets, AKT1 was identified as a core hub target. Considering the reported role of AKT activation in controlling HIF‐1*α*‐dependent VEGFA expression, western blotting was subsequently performed to examine proteins associated with the AKT/HIF‐1*α* signaling axis. The model group exhibited decreased p‐AKT/AKT ratios and lower HIF‐1*α* and VEGFA protein levels, whereas ZLO treatment partially reversed these molecular changes (Figure 7A–D).

## 4. Discussion

Administration of ZLO improved the healing outcome of diabetic wounds in db/db mice, suggesting its potential role in promoting wound repair. Given the central roles of persistent inflammation and impaired angiogenesis in diabetic wound pathology [27, 28], we next considered how ZLO affected these key aspects of tissue repair. During the early postinjury period, all experimental groups, including the ZLO‐treated group, exhibited a transient increase in wound area within the first 7 days. Similar early wound expansion has been reported in diabetic wound models [26]. This phenomenon may partly result from excessive exudate accumulation, which can promote periwound maceration and increase vulnerability to mechanical injury [29]. These pathological changes may further exacerbate local inflammation, as evidenced by the sustained elevation of proinflammatory cytokines, including IL‐6 and TNF‐*α*, during the early phase of wound healing [30]. Importantly, ZLO treatment significantly attenuated wound enlargement compared with untreated diabetic controls. Several factors may collectively contribute to this improvement, including the intrinsic pharmacological properties of ZLO, the barrier and hydration effects provided by the Vaseline base, and the exudate‐handling capacity of the dressing.

Histological analyses showed that ZLO enhanced re‐epithelialization, granulation tissue formation, and collagen deposition. Consistent with these histological improvements, ZLO treatment increased the expression of angiogenic markers CD31 and VEGFA and promoted macrophage polarization toward an M2‐like phenotype, suggesting coordinated regulation of vascular regeneration and local immune responses. Consistently, reduced TNF‐*α* and IL‐1*β* levels in wound tissues further indicated that ZLO alleviated local inflammation. Together, these findings indicate that ZLO coordinates tissue regeneration, angiogenesis, and inflammatory regulation during diabetic wound healing. Although *α*‐SMA expression was slightly increased in the ZLO group compared with controls, no statistically significant difference was observed. This trend may be related to differences in healing stage progression, as control wounds may have progressed further into the remodeling phase, during which myofibroblast activity and *α*‐SMA expression typically decline.

KEGG enrichment analysis suggested that the HIF‐1 signaling pathway may be involved in ZLO‐mediated diabetic wound repair, whereas PPI network analysis identified AKT1 as a core hub target. Based on these complementary findings, we selected AKT/HIF‐1*α*‐associated signaling for further experimental investigation. HIF‐1*α* is an important regulator of epithelialization, angiogenesis, granulation tissue formation, and macrophage polarization; however, under diabetic conditions, persistent hyperglycemia may impair its stability and downstream activity [31, 32]. AKT activation has been reported to enhance HIF‐1*α* stability and facilitate metabolic adaptation to hypoxic stress [33, 34].

Given the close association between HIF‐1*α* and glycolytic regulation, we further examined the expression of GLUT1, PKM2, and LDHA. These proteins regulate glucose uptake, glycolytic flux, and lactate production, and PKM2 additionally participates in HIF‐1*α*‐dependent transcriptional and inflammatory regulation [35–41]. Consistent with these roles, immunohistochemical analysis showed higher expression levels of GLUT1, PKM2, and LDHA in wound tissues compared with adjacent normal skin, suggesting increased glycolysis‐associated metabolic activity during wound repair. GLUT1 staining was predominantly cytoplasmic, with occasional membrane localization, which may reflect dynamic intracellular trafficking and increased glucose transport in metabolically active cells [42]. PKM2 exhibited both cytoplasmic and nuclear localization in a subset of cells, consistent with its dual roles in glycolytic metabolism and the transcriptional regulation of wound‐healing‐related genes.

Interestingly, several glycolysis‐associated proteins did not differ significantly between the control and model groups at Day 14 after injury. This may partly reflect the advanced remodeling stage of the control wounds, during which metabolic activity declines after the completion of active tissue repair, thereby narrowing the difference between the two groups. In contrast, ZLO treatment restored HIF‐1*α* expression and increased GLUT1, PKM2 and VEGFA levels, suggesting sustained metabolic and angiogenic activity associated with ongoing tissue repair. Notably, ZLO did not further increase LDHA expression, indicating that its beneficial effects may involve selective metabolic remodeling rather than global activation of glycolysis. Western blotting further demonstrated restored HIF‐1*α* expression, enhanced AKT phosphorylation, and increased VEGFA expression following ZLO treatment.

Overall, ZLO promoted diabetic wound healing by attenuating inflammation, enhancing collagen deposition, and promoting angiogenesis, potentially involving AKT/HIF‐1*α*‐associated signaling and glycolytic regulation. However, the glycolytic findings were based primarily on protein expression without functional, cell‐specific, or pathway‐specific validation. In addition, this study relied on a single db/db mouse model, which may not fully reproduce the clinical heterogeneity of human diabetic wounds. The limited sampling time points may also have restricted assessment of the temporal dynamics of wound repair. In addition, the contribution of individual constituents within the ZLO formulation to its biological effects remains unclear and warrants further investigation. Future studies using additional animal models, cellular and functional experiments, and component‐specific validation are needed to clarify these mechanisms and further evaluate the therapeutic potential of ZLO.

**Nomenclature**
DL: drug‐likeness
GO: Gene Ontology
H&E: hematoxylin and eosin
KEGG: Kyoto Encyclopedia of Genes and Genomes
LC–MS: liquid chromatography–mass spectrometry
PPI: protein–protein interaction
TCM: traditional Chinese medicine
WB: Western blot
ZLO: Zilian ointment

## Author Contributions

Cheng Qin: writing—original draft, formal analysis. Yiwen Zhang: investigation, formal analysis. Duhua Chen: investigation. Jin Xu: visualization. Jing Yang: validation. Fei Liu: supervision. Qin Li: project administration. Xiang Li: supervision, methodology, writing—review and editing. Jianzhou Ye: conceptualization, supervision, writing—review and editing. Xuesong Yang: funding acquisition, conceptualization, supervision, writing—review and editing. Cheng Qin and Yiwen Zhang contributed equally.

## Funding

This work was supported by the Yunnan Provincial Department of Science and Technology Project (202401AZ070001‐014 and 202302AA310014).

## Disclosure

All authors have read and approved the final version of the manuscript. Xuesong Yang had full access to all of the data in this study and takes complete responsibility for the integrity of the data and the accuracy of the data analysis.

## Ethics Statement

All animal experimental procedures were reviewed and approved by the Experimental Animal Ethics Committee of Yunnan Provincial Hospital of Traditional Chinese Medicine (Approval No. DW‐2025‐036).

## Conflicts of Interest

The authors declare no conflicts of interest.

## Supporting information


**Supporting Information** Additional supporting information can be found online in the Supporting Information section. Table S1: Provides detailed information on the 225 overlapping genes identified by intersecting the predicted targets of the active components of ZLO with diabetic wound‐related genes.

## Data Availability

The data that support the findings of this study are available from the corresponding author upon reasonable request.
