# Supplementary material for: Zilian Ointment Promotes Diabetic Wound Healing via Akt/HIF‐1α–Mediated Angiogenesis
Source: J Diabetes Res. 2026 Sep 24;2026:9928022. doi: 10.1155/jdr/9928022 (PMC13601937; doi:10.1155/jdr/9928022)
Supplement: Supplementary file 1 [file JDR-2026-9928022-s001.docx]

**Supplementary Table. S1** Intersection of active ingredient targets and diabetic wound genes.

| 1 | 2 | 3 | 4 | 5 | 6 | 7 | 8 | 9 | 10 |
| --- | --- | --- | --- | --- | --- | --- | --- | --- | --- |
| CASP3 | CDK4 | BCL2L1 | NFKBIA | JUN | PRKCD | AHR | CLU | ESR2 | GPX1 |
| BCL2 | PTGS2 | BIRC5 | AR | LRP5 | PRSS1 | AKR1B10 | CMA1 | F10 | GZMB |
| TP53 | TNF | CASP9 | ATM | MAPK1 | PTGS1 | ALB | COL6A1 | F12 | HGF |
| BAX | AKT1 | CDK2 | CDKN2A | MAPK3 | PTPN6 | ATP6V1E1 | CORIN | F2 | HP |
| MMP2 | CASP8 | CTNNB1 | CXCL8 | MYC | RELA | C1S | CTSD | F2RL1 | HSPA5 |
| CDKN1A | CDKN1B | CYP2C19 | CYP1A2 | NOS1 | RHOA | CAV1 | CTSG | F7 | INPPL1 |
| CYP1A1 | GFAP | ESR1 | CYP3A4 | NR1I2 | VEGFA | CCL2 | CYCS | F9 | INS |
| IL6 | NOS2 | FOS | FN1 | PARP1 | ABCB1 | CFB | DDIT3 | FAS | ITGA6 |
| MMP9 | NOS3 | HMOX1 | GSK3B | PLAU | ACSL1 | CFI | DGAT1 | FASLG | ITGAM |
| CCND1 | STAT3 | LRP1 | GSTP1 | PPARG | ACTA1 | CHEK2 | ELANE | FOXO1 | ITPR1 |
| KLK1 | NQO1 | SLC2A4 | AIFM1 | CAPN1 | CREB1 | ERN1 | HRAS | MAPK8 | NR1H3 |
| KRAS | PIK3C3 | SMAD3 | AKT3 | CASP7 | CSF2 | FADD | ICAM1 | MIR148A | NR3C1 |
| LPA | PKM | TGFB1 | ALDH1A1 | CAT | CXCR4 | FGF2 | IFNA2 | MIR21 | NRAS |
| MACF1 | PLAT | TMPRSS6 | ANXA5 | CCL17 | CYP2C9 | FOXO3 | ILK | MMP10 | NTS |
| MAPK14 | PLG | TNFSF10 | APOC1 | CCR3 | CYP2D6 | GBA1 | IRS1 | MPO | PECAM1 |
| MAPT | PROC | VDAC1 | ATF6 | CD44 | DNMT1 | GSR | JAK1 | MTOR | PIK3CA |
| MMP1 | PRTN3 | VIM | BAMBI | CDC42 | EP300 | HBEGF | JAK2 | MYD88 | POU5F1 |
| MMP7 | RAF1 | ACTA2 | BDNF | CDH1 | EPCAM | HK2 | KDR | NANOG | PPARA |
| NES | ROCK1 | ADIPOQ | BMP4 | CLDN1 | ERBB2 | HMGB1 | KEAP1 | NFE2L2 | PPARD |
| NFKB1 | ROCK2 | ADRB2 | BRCA1 | COL1A1 | ERBB3 | HNF4A | LPL | NPPA | PRL |
| PTPN1 | PTPN2 | PTX3 | PXDN | RAC1 | RAC2 | RPS6KB1 | RRAD | RXRA | SERPINE1 |
| SIRT3 | SMAD7 | SPARC | SRC | THRB | TIMP2 | TLR4 | TPO | TRPV4 | TUBB3 |
| TWIST1 | UGT1A1 | WNT5A | WT1 | XBP1 |  |  |  |  |  |
